# Costs of Testing for Ocular *Chlamydia trachomatis* Infection Compared to Mass Drug Administration for Trachoma in The Gambia: Application of Results from the PRET Study

**DOI:** 10.1371/journal.pntd.0003670

**Published:** 2015-04-22

**Authors:** Emma Harding-Esch, Mireia Jofre-Bonet, Jaskiran K. Dhanjal, Sarah Burr, Tansy Edwards, Martin Holland, Ansumana Sillah, Sheila West, Tom Lietman, Jeremy Keenan, David Mabey, Robin Bailey

**Affiliations:** 1 London School of Hygiene and Tropical Medicine, London, United Kingdom; 2 Public Health England, HIV/STI Department, London, United Kingdom; 3 Department of Economics, City University, London, United Kingdom; 4 Abacus International, Bicester, Oxfordshire, United Kingdom; 5 MRC Laboratories, Fajara, The Gambia; 6 National Eye Health Programme, Banjul, The Gambia; 7 Dana Center for Preventive Ophthalmology, Wilmer Eye Institute, Johns Hopkins University, Baltimore, Maryland, United States of America; 8 The Francis I. Proctor Foundation, University of California, San Francisco, San Francisco, California, United States of America; University of California San Diego School of Medicine, UNITED STATES

## Abstract

**Background:**

Mass drug administration (MDA) treatment of active trachoma with antibiotic is recommended to be initiated in any district where the prevalence of trachoma inflammation, follicular (TF) is ≥10% in children aged 1–9 years, and then to continue for at least three annual rounds before resurvey. In The Gambia the PRET study found that discontinuing MDA based on testing a sample of children for ocular Chlamydia trachomatis(*Ct*) infection after one MDA round had similar effects to continuing MDA for three rounds. Moreover, one round of MDA reduced disease below the 5% TF threshold. We compared the costs of examining a sample of children for TF, and of testing them for *Ct*, with those of MDA rounds.

**Methods:**

The implementation unit in PRET The Gambia was a census enumeration area (EA) of 600–800 people. Personnel, fuel, equipment, consumables, data entry and supervision costs were collected for census and treatment of a sample of EAs and for the examination, sampling and testing for Ct infection of 100 individuals within them. Programme costs and resource savings from testing and treatment strategies were inferred for the 102 EAs in the study area, and compared.

**Results:**

Census costs were $103.24 per EA plus initial costs of $108.79. MDA with donated azithromycin cost $227.23 per EA. The mean cost of examining and testing 100 children was $796.90 per EA, with Ct testing kits costing $4.80 per result. A strategy of testing each EA for infection is more expensive than two annual rounds of MDA unless the kit cost is less than $1.38 per result. However stopping or deciding not to initiate treatment in the study area based on testing a sample of EAs for Ct infection (or examining children in a sample of EAs) creates savings relative to further unnecessary treatments.

**Conclusion:**

Resources may be saved by using tests for chlamydial infection or clinical examination to determine that initial or subsequent rounds of MDA for trachoma are unnecessary.

## Author Summary

Trachoma, caused by infection with a bacterium (chlamydia) is controlled by mass drug administration (MDA), which is recommended yearly for districts in which a trachoma problem has been found to exist. The decision, after several rounds, that MDA is no longer needed is currently based on clinical signs of trachoma, but these are an unreliable indicator of infection. The PRET study, in the Gambia, found that tests for infection could be used to show that subsequent rounds of MDA were redundant. This paper shows, by estimating the costs, that testing children in a sample of census districts for infection can save resources compared to (unnecessary) rounds of MDA.

## Introduction

Trachoma, caused by ocular infection with *Chlamydia trachomatis (Ct)*, is the leading infectious cause of blindness worldwide and is estimated to cause 3.6% of the world’s blindness [1]. The presence of follicles and inflammation in the upper tarsal conjunctiva, known as active trachoma, is characteristic of childhood infection. Following years of repeated infection, the upper tarsal conjunctiva may become so severely scarred that the eyelashes turn inwards, rub on the eyeball and cause corneal opacity and blindness.

The World Health Organization (WHO) estimates that worldwide, 40.6 million people have active trachoma, 8.2 million people have in turned eyelashes (trichiasis), and 1.3 million are blind as a result of trachoma [1,2]. It was estimated in 1995 that $2.9 billion is lost in annual revenue as a result of the loss of vision arising from trachoma[3]. Trachoma is most prevalent in poor, rural communities with low standards of hygiene and sanitation. It is thought to be endemic in 57 countries [2].

The WHO recommendations for the control and elimination of trachoma are based on a strategy with the acronym “SAFE”: **S**urgery for in turned eyelashes, **A**ntibiotics to treat ocular *Ct* infection, **F**acial cleanliness and **E**nvironmental improvement to reduce transmission of the infection. The WHO recommends that mass treatment with an antibiotic such as azithromycin should be given annually to districts or communities where the prevalence of follicular trachoma (TF) is ≥10% in children aged 1–9 years, continuing for at least three rounds before the need to re-survey. In some settings however, including the Jareng village cluster in The Gambia [4] and Rombo district in Tanzania [5], a single round of high coverage mass azithromycin reduced *Ct* infection to very low and unsustainable levels, although the prevalence of TF would still have indicated that intervention was needed. Testing for *Ct* demonstrated that further treatments were unnecessary.

In 2007, The Gambia implemented a national plan for trachoma control [6] with a donation of azithromycin through the International Trachoma Initiative. In this plan, based on a 2006 survey [7], extrapolation and local knowledge, 11 districts demonstrated or believed by the programme to have a prevalence of TF greater than 10% in 1–9 year olds were assigned to mass drug administration (MDA) with azithromycin. The Partnership for the Rapid Elimination of Trachoma (PRET) study [8,9] aimed, *inter alia*, to test whether one round of MDA would be sufficient to control active trachoma across a wide geographical area, comprising four of the eleven districts assigned to MDA and containing 67,156 people. The study compared communities randomised to receive yearly MDA for three years or to a stopping rule (SR) in which mass treatment would cease if the estimated prevalence of either TF or *Ct* infection at six months were sufficiently low. A further stopping rule was applied at the district level, according to which treatment in non-study communities would also cease if the district prevalence of infection or of TF were sufficiently low,. In the study, the TF prevalence in 0–5 year-olds in the study area was reduced below 3% after one round of treatment and *Ct* infection, which was at a low level initially, was not detectable in any child at 12 and 18 months of follow up. The study found no difference in outcome (TF or *Ct* infection at 36 months) between the stopping rule communities and those in which treatment continued [8], illustrating that tests for *Ct* infection (or clinical examination for TF) could be used to demonstrate that initial or subsequent rounds of MDA were redundant.

Data were gathered during PRET The Gambia with the aim of comparing the programme costs of implementing a stopping rule based on tests for infection with those of further rounds of treatment and to explore the situations in which testing for infection would have a cost advantage over two further treatments. We report on these cost data and on their application to this and other possible testing and treating strategies.

## Methods

### PRET study

**Fig 1:**
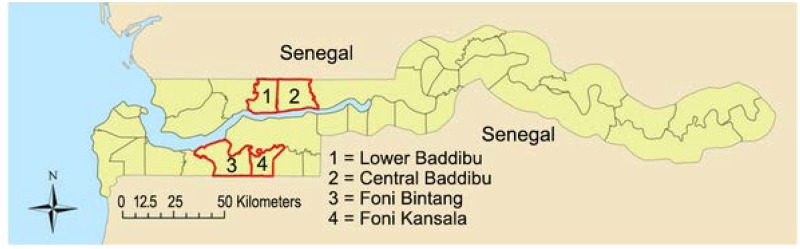
Map of The Gambia showing location of the study area and 4 study districts.

The study was conducted from 2008 to 2011 in the Foni Bintang and Foni Kansala districts in Western Region, and in Central Baddibu and Lower Baddibu in North Bank Region. These are shown on the map (Fig 1). For census purposes, The Gambia is divided into geographically defined census Enumeration Areas (EAs), of similar population size, notionally containing 600–800 people. An EA is a useful unit for representative sampling, as randomly choosing EAs is equivalent to sampling settlements with probability proportional to their size. EA geography varies in ways which might influence costs; an EA is either a segment of a large settlement (segment) a single medium-sized settlement (single), or made up of multiple adjacent small settlements (multiple).

### Randomisation

The randomisation scheme in PRET has been described previously [8,9]. Briefly, all 102 EAs were randomly assigned to one of four arms: 1) standard treatment coverage, SR; 2) standard treatment coverage, 3 annual MDAs; 3) enhanced treatment coverage, SR; 4) enhanced treatment coverage, 3 annual MDAs; under the restriction that each settlement was treated in the same way (all EAs representing segments of the same settlement were in the same arm). The outcomes were assessed in a random selection of 48 EAs for sampling, which was made such that 12 EAs per arm and per district were selected (3 EAs per arm per district) and such that each large settlement was represented by only one of its segment EAs. This ‘sample’ of 12 EAs per district then served as the basis for implementing district-level stopping rules in those EAs in the district not included in the sampling.

### Field and laboratory work

Details of PRET in The Gambia have been described elsewhere [8–10]. Briefly, all members of every household in the 48 EAs selected for sampling were listed in a census and a random sample of 100 children aged 0–5 years per EA had both eyes examined for the clinical signs of trachoma using the WHO simplified grading system [11]. An ocular swab was then taken for detection of ocular *Ct* infection by Amplicor Polymerase Chain Reaction (PCR) (Roche Molecular Systems, Branchburg, NJ, USA), as previously described [12]. A new random sample of 100 children aged 0–5 was examined in each EA at 6, 12, 18, 24, 30 and 36 month follow-up time points.

Samples were processed by two laboratory technicians at Medical Research Council (MRC) Laboratories, The Gambia, by Amplicor PCR. The manufacturer’s instructions were followed, except for sample preparation where a previously published method was used [12], and the extracts of five swabs were pooled, with individual testing of any positive pools.

### MDA in PRET

At baseline, all 102 EAs in the four districts were mass treated with azithromycin by the Gambian National Eye Health Programme (NEHP). EAs assigned to standard treatment coverage were visited on a single day, whereas those assigned to enhanced coverage were visited a second day to treat those not treated on the first visit. The treatment teams were kept unaware, on their first visit to an EA, of the coverage assignments. Children were dosed using height sticks, with cut-offs optimally derived from local height/weight data to minimise the risk of over- and under-dosing [6,13]. EAs were usually treated by a team of six people, working in three pairs, plus a driver. In segment EAs, all six team members worked together whereas in multiple EAs pairs would work on their own in the different settlements. In each pair, one measured the height and distributed the treatment, while the other recorded the treatment information against the census in the treatment book.

### Stopping rule in PRET

Under the stopping rule, the decision to treat at 12 months post-baseline was based on the clinical examination and ocular *Ct* infection data at 6 months (Table 1). MDA was discontinued in study EAs in the SR arms if there were either no cases of *Ct* infection or no cases of TF in the 100 sampled individuals (equivalent to 95% confidence that the true prevalence was less than 5%). Furthermore, MDA was discontinued throughout a district (excluding those EAs randomised to three annual treatments) if, based on the EAs that were sampled, there was 95% confidence that the prevalence of infection, (or of TF) in the district was below 5%.

**Table 1:** Prevalence of TF and ocular *C*. *trachomatis* infection in PRET by allocation.

	3 annual MDAs		Stopping rule (MDA at baseline only)		Standard coverage (one treatment visit)		Enhanced coverage (extra treatment visit)	
Time point	TF (%)	*Ct* (%)	TF (%)	*Ct* (%)	TF (%)	*Ct* (%)	TF (%)	*Ct* (%)
**Baseline**	**6.5**	**0.8**	**6.1**	**0.6**	**5.8**	**0.4**	**6.8**	**1.1**
**6 months**	**2.4**	**0.1**	**2.4**	**0**	**2.2**	**0**	**2.5**	**0.1**
12 months	2.6	0	2.7	0	2.6	0	2.7	0
18 months	1.6	0	1.7	0	1.9	0	1.4	0
24 months	2.3	0	2.1	0.1	2.4	0.04	2.1	0.04
30 months	2.4	0.4	3.6	0.1	2.8	0.2	3.2	0.3
**36 months**	**2.6**	**0.6**	**2.9**	**0.4**	**2.4**	**0.6**	**3.1**	**0.4**

### Cost data collection

Cost data, which included personnel, fuel, equipment, consumables, data entry and supervision, were collected for census, sampling and examination. Treatment cost data were collected 12 months post baseline from the 12 EAs assigned to the enhanced coverage treatment arm, with the costs for one day of treatment (standard coverage) inferred by removing the costs of the second day from the total EA treatment cost. Examination cost data were collected from these same EAs at 18 months post baseline. Efforts were made to identify and exclude the costs of concurrent research activity, such as personnel and consumables involved in taking eyelid photographs, and completing consent forms.

As is recommended for cost studies [14], worksheets detailing all costs involved for the day’s activities(examination or treatment) were completed each day. Unit costs were obtained from local sources when available, and when not, the original source price was taken. The laboratory cost of processing the samples was calculated using information provided by the MRC Laboratories, The Gambia. Costs were obtained in US Dollars ($), British Pounds (GBP), and Gambian Dalasi (GMD). GMD and GBP costs were converted to $ using a historic currency conversion of an average of 366 days from the 1^st^ January 2009 to the 1^st^ January 2010 (http://www.oanda.com/currency/historical-rates/). For this time period, 1GMD = $0.0377, and 1GBP = $1.5665. Cost data were entered and analysed in Microsoft Excel 2007.

### Personnel

Salaries, including overheads, were converted to a daily rate. For the census, it was assumed that NEHP staff would first attend a training workshop, and that subsequently one census taker, using a motorcycle, could census one EA/day. For the treatment team, per diems to cover food and accommodation in the field were given as a single payment based on the expected number of days needed to complete the treatment. The team’s total per diem was divided by the number of days worked to obtain a daily rate. The PRET field team contained research workers and NEHP Community Ophthalmic nurses (CONs). For costing we assumed that three NEHP CONs (for form filling, grading and the field lab) and a NEHP driver would undertake the work. Examination team received per diems for each day worked. Volunteers from the communities who facilitated field work were also compensated for their time and effort. For the laboratory, personnel costs included the salaries of two lab technicians, employed locally by the MRC Laboratories, who spent 85% of their time processing the samples. Based on an average of 920 samples being processed a week, a lab personnel cost per sample processed was calculated.

### Fuel

The cost of fuel was calculated based on the distances in kilometres (km) travelled by the teams as read from the vehicle dashboards and the refuelling costs.

### Equipment

Equipment for the treatment team included the vehicle, height sticks and weighing scales. Equipment for the examination team included the vehicle, table, chairs and loupes. Laboratory costs were obtained for all equipment necessary to run Amplicor PCR. The equipment cost/day was calculated by dividing the capital cost, by the equipment’s life expectancy in years multiplied by 345 (number of assumed working days per year).

### Consumables

For the census, consumable costs of clipboards, pens, phone credit and stationery were included. For treatment, consumables included the cost of medication. Azithromycin for trachoma control is donated free to the NEHP and to other trachoma control programmes by the International Trachoma Initiative, but storage and transport costs are met by the programmes and were included. The costing gold standard [14] of taking into account the opportunity cost (i.e. when goods or services are donated, a ‘replacement’ cost is imputed) was applied (drugs were assumed purchased rather than donated). In this case we assumed the online market rates of $20 for 30x250mg azithromycin tablets, and $9 for a bottle of 30 ml paediatric oral suspension. Costs of tetracycline eye ointment, which is purchased and offered to children aged under 6 months and pregnant women in MDA campaigns were included. For examination, field consumables included swabs, tubes, labels, paper, gloves, waste bags, ointment, torches and batteries, phone credit and stationery. For the laboratory, costs of all consumables necessary for processing samples by Amplicor were included, together with the cost of kits.

### Test kit costs

We define the Amplicor test ‘kit cost’ as the amount spent on Amplicor kits to generate one test result using a strategy of testing in pools of five and retesting all positive or equivocal pools.

### Data entry

We assumed the NEHP would enter data rather than using the PRET research data entry system. We assumed that one data entry clerk would be employed, entering two EAs per day for treatment data, and four EAs per day for examination data.

### Supervision

We assumed that the NEHP manager (a senior civil servant) would spend one day per week supervising the census, and two days per week in the field to supervise the treatment and examination teams (one day per team). Supervision costs included the manager’s salary, vehicle (car depreciation) and fuel, calculated as outlined above. For the laboratory, we assumed that locally employed technicians were supervised by a Scientific Officer (a scientist with a Master’s degree employed by the MRC on a sub-regional salary scale) at 5% of their time.

### Cost calculations

The treatment and exam cost data were recorded from twelve EAs (five multiple, two single and five segments) at the 12 and 18 month time points respectively. Census costs were estimated from records of training workshops and field records at baseline. Total EA level costs were calculated by summing the personnel, fuel, equipment, consumables, data entry and supervision costs. For both examination and treatment, when more than one EA was visited in a day, the number of individuals treated, or number of children examined, in the EA was used to provide a weighted cost per EA for items that were not “per individual/child” (personnel, fuel, and equipment costs). Summary estimates were made for each type of EA and then extrapolated for the study sample (48 EAs), and for the whole study area (102 EAs), according to their decomposition by EA type. Results are expressed as total costs in US dollars ($) over the study area, costs per EA and costs per head of the population of the study area.

### Ethical approval

The study was conducted in accordance with the declaration of Helsinki. Written informed consent was given by adult subjects or by the parent or guardian of child participants. Ethical approval was obtained from the London School of Hygiene & Tropical Medicine (LSHTM), UK, Ethics Committee and The Gambia Government/Medical Research Council (MRC) joint Ethics Committee, The Gambia.

## Results

### PRET study[8]

**Fig 2:**
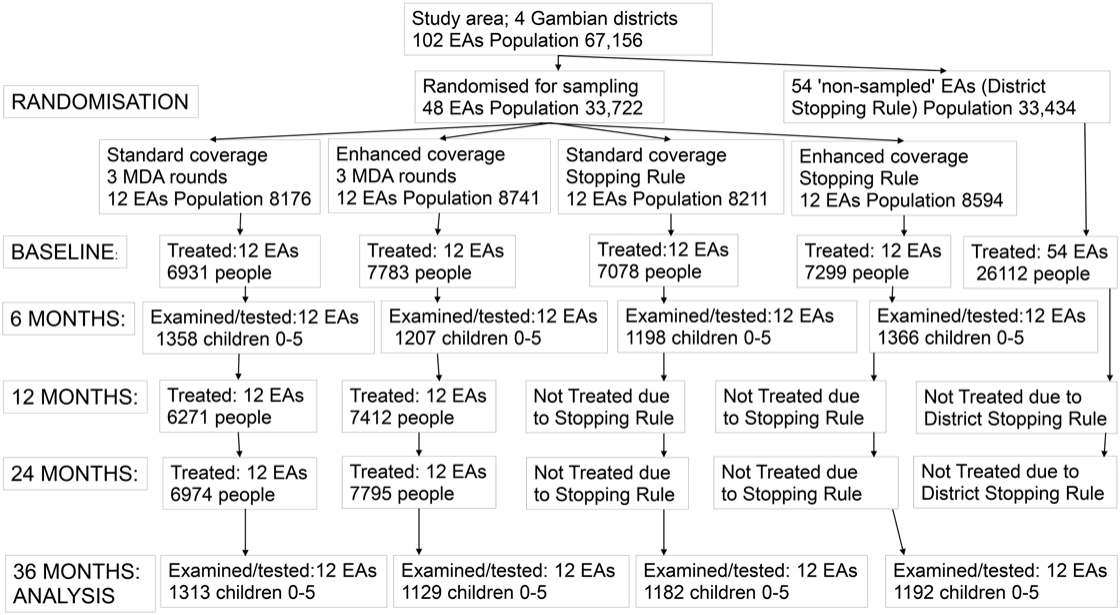
Summary of PRET study illustrating randomisations, target populations and effects of stopping rules based on examination and testing after one MDA round.

Fig 2 illustrates the target populations, randomisations, study arms, MDA treatment rounds and effects of the stopping rules (based on examining and testing 100 children per sampled EA 6 months after the first MDA round in the PRET study. The mean and median baseline EA populations in the 48 sampled EAs were 701 and 667 respectively. The mean and median baseline EA populations in the study area of 102 EAs were 658 and 622 respectively. The baseline TF prevalence was 6.5% of 0–5 year olds. Six months after the baseline mass treatment, TF prevalence was 2.4% (95% CI 1.6–3.1) and no *Ct* infection was found in any of the 24 EAs randomised to the stopping rule. Implementing the stopping rule led to mass treatments being discontinued in these 24 EAs. Furthermore, implementing the district stopping rule led to treatment being discontinued in all 54 non-study EAs across all four districts. MDA only continued in the 24 EAs randomised to three annual treatments, where MDA at 12 months and 24 months was implemented regardless of the prevalence of TF or of infection. At baseline, the prevalence of *Ct* infection was 0.8% (95% CI 0.3–1.2). On average, enhancing coverage by making an extra visit improved MDA coverage of 0–9 year-olds by 3%(from 90.2% to 93.2%).[8] At 36 months there were no differences in the study outcomes between the study arms (Table 1). Specifically, neither enhanced coverage nor the two additional mass treatments in the 3x annual treatment arm had any effect on TF or infection prevalence at 36 months. There were also no differences at intermediate time-points. Thus, a single round of MDA reduced TF to low levels, and there were no apparent benefits to two further rounds of mass treatment, relative to discontinuing MDA post-baseline based on tests for infection[8].

### Alternative sampling scenario calculations

In PRET 48 EAs were sampled and 100 children 0–5 per EA examined and tested for infection at each time point. This was because the PRET study explored a stopping rule in each EA, necessitating almost all the eligible children aged 0–5 being examined in order to have 95% confidence(with zero observed cases) that the underlying prevalence of TF or of Ct infection in the EA was less than 5%. For a stopping rule applied to the whole study area a smaller number of sampled EAs would have sufficed for 95% confidence that the underlying area-wide prevalence of TF or Ct infection were less than 5%. We used recommendations for sampling surveys in the WHO manual for trachoma programme managers [15] to estimate the number of EAs that would have been needed to be sampled, examined and/or tested in order to determine a stopping rule of 95% confidence TF or Ct infection prevalence less than 5% across the study area of 102 EAs, given the prevalences observed in the PRET sample. These calculations were based on the design effects [16] estimated in EA summarised data from PRET at baseline and 6 months [8].

### Costs

Costs are presented per EA and then applied as estimates to the study area of 102 EAs and 67,156 people according to decomposition by EA type as outlined above and in Tables 2,3,and 4.

**Table 2:** Census costs by item.

Item	Cost (USD)
**Training (fixed):**	
Daily salary of CON for 2 days	11.63
Daily salary of NECP manager for 2 days	74.545
Per diem of CON for 2 days (assume CON displaced from home)	22.62
**Total training cost**	**108.79**
**Cost per EA:**	
**Personnel**	
CON monthly salary Multiplied by 1.2 for overheads and divided by 22 for daily salary	5.71
CON per diem (assume CON not displaced from home)	7.54
**Materials**	
Stationery (Pens & Clipboard: 1/census taker)	1.89
Paper (average number of households per EA: 65; average number of people in EA: 712; therefore average number of people per household: 11, which could fit onto one piece of paper): 65 sheets per EA)	0.50
Plastic wallet (1/EA)	0.56
Phone credit (D50/week)	0.38
**Transport**	
Motorcycle depreciation (€1,735 cost + (£2,050 freight charges + £230 insurance for shipment of 7 bikes)). Life expectancy = 5 years Cost/day = cost of motorbike / (345 x life expectancy)	1.52
Fuel (D32/litre, assume 20km /litre). EA fuel cost = number of km x 32 / 20. Average number of km = 71.8km (based one just one visit to each EA)	4.33
**Data entry** (2 EAs/day, 1 NECP enterer) Average cost per EA of data entry = 0.5*(salary * 1.2 (overheads) /22)	3.08
**Supervision**	
Salary (Salary of NECP manager for 1 day/week, divide by 22)	37.37
Vehicle depreciation (Depreciation cost of vehicle: $49,900 + D23,000 insurance, licence & road tax.	16.84
49,900/(345x10) = $14.464 = cost/day of vehicle	
D23,000 x 0.0377 = $867.1	
$867.1 / 365 = $2.376/day of tax, licence etc.	
$2.376 + $14.464 = total cost of vehicle per day	
Fuel (to go to field one day/week): Average distance travelled to go into field for one day = 195km from Banjul to Farafenni.	23.52
**Total per EA cost**	**103.24**
**Cost for 102 EAs**	**10,530.48**
**Total census cost for study area**	**10,639.27**

Assumptions:
Costs were obtained in US Dollars (USD), British Pounds (GBP), and Gambian Dalasi (GMD). GMD and GBP costs were converted to USD using a historic currency conversion of an average of 366 days from the 01st January 2009 to the 1st of January 2010 (http://www.oanda.com/currency/historical-rates/). For this time period, 1GMD = 0.0377 USD, and 1GBP = 1.5665 USD. For training, the following assumptions were made:
Two days’ training. Training was done at the Regional Eye Care Centre, so there are no facility costs. Training was done by the manager of the NECP, who has no per diem.
For census taking, the following assumptions were made:
One NECP census takers on a motorcycle per EA One census taker can census 1 EA/day (based on PRET) The census taker would not do a first separate trip to make a household head list

**Table 3:** Costs of MDA rounds.

	EAs made up of smaller settlements (M-multiple)		EAs made up of one settlement (S-single)		EAs part of a large settlement (G-segment)		Weighted average across 48 study EAs: 25M 7S 16G		Weighted average across 102 EAs in the study area: 31M 11S 60G	
Costing category	Standard	Enhanced	Standard	Enhanced	Standard	Enhanced	Standard	Enhanced	Standard	Enhanced
Personnel	109.4	168.3	94.7	106.4	136.4	204.3	116.26	171.27	123.70	182.80
Fuel	6.3	10.2	3.7	4.1	12	15.7	7.82	11.14	9.37	12.78
Equipment	9.3	14.8	8.3	9.3	10.5	15.8	9.55	14.33	9.90	14.80
Supervision	77.8	77.8	77.8	77.8	77.8	77.8	77.8	77.8	77.8	77.8
Data entry	3.1	3.1	3.1	3.1	3.1	3.1	3.1	3.1	3.1	3.1
Tetracycline	2.8	3.5	1.7	2.2	4.8	5.9	3.31	4.11	2.98	4.77
**Average total cost/EA, excluding azithromycin**	**208.7**	**277.7**	**189.3**	**202.9**	**244.6**	**322.6**	**217.84**	**281.76**	**227.73**	**296.05**
Azithromycin	1658.6	1874.3	945.5	1195.1	1088.6	1192.9	1364.61	1548.12	1246.40	1400.23
**Average total cost/EA, including azithromycin**	**1867.3**	**2152**	**1134.8**	**1398**	**1333.2**	**1515.5**	**1582.44**	**1829.88**	**1474.13**	**1696.27**

**Table 4:** Estimated examination and testing cost per EA in the study EAs and across the study area, by costing category (USD).

Costing category	Average cost per EA (USD)				
EA type:	Multiple(M)	Single(S)	Segment(G)	48 study sample EAs adjusted for EA type(16M 7S 25G)	102 study area EAs adjusted for EA type(31M 11S 60G)
Field personnel	58	121.9	78.7	74.22	77.07
Field consumables	58.4	61.1	58.2	58.73	58.57
Field equipment	9.7	18.1	12.7	11.93	12.37
Field supervision	77.8	77.8	77.8	77.8	77.8
Fuel	16.7	12.8	17.1	16.26	16.51
Lab personnel	20.6	21.5	20.5	20.70	20.64
Lab consumables	43.8	45.8	43.6	44.03	43.90
Lab kit (Amplicor)	479	500.6	477	481.48	480.15
Lab equipment	6.6	6.9	6.5	6.61	6.57
Lab supervision	1.8	1.9	1.8	1.81	1.81
Data entry	1.5	1.5	1.5	1.5	1.50
**Estimated total cost/EA**	**773.9**	**869.9**	**795.4**	**795.07**	**796.90**
**Estimated total cost/head of population**	1.176	1.322	1.209	1.21	1.21

### Census costs

The breakdown of census costs is shown in Table 2. The cost of the census was $108.79 for training plus $103.24 per EA. The estimated cost of census in the study area was $10,639.27. *Per diem* costs were the greatest component of training, and supervision costs the greatest component of the census.

### Treatment costs

**Fig 3:**
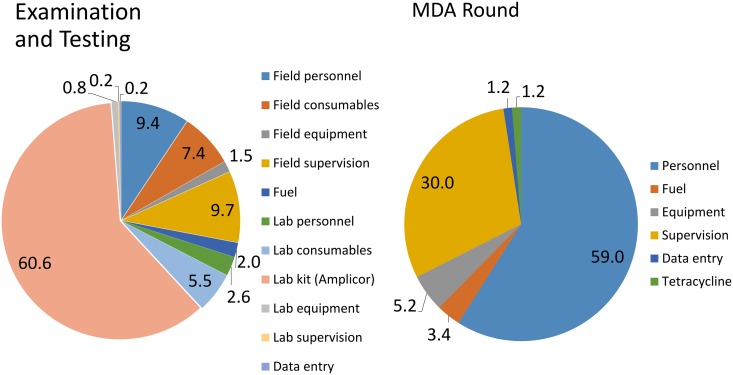
Pie charts illustrating relative component costs for treatment, examination and testing.

Over the study area the estimated average cost of one MDA round in an EA was $227.73 for standard treatment and $296.05 for enhanced treatment (Table 3). The estimated cost of a standard round of MDA in the study area(102 EAs) was $23,228.46, or $0.35 per head of target population, increasing to $0.39 per head for the extra visit in the enhanced treatment. Personnel represented the greatest treatment cost, followed by supervision (Fig 3). Treatment costs varied slightly depending on the geography of the EA, with single EAs being cheapest to treat and segments the most expensive (data in Table 2).

### Examination costs

Over the study area the average EA cost of examining and testing 100 children was $796.90. As anticipated costs varied by EA geography: and were $773.90, $869.90 and $795.40 in multiple, single and segment EAs respectively (Table 4). The major laboratory cost was the Amplicor kit cost, at $480.15 for 100 samples per EA (Fig 3). The major field costs were personnel and supervision (Fig 3), whose relative importance varied depending on the geography of the EA (data in Table 4). Examining and testing all EAs in the study area, adjusting for EA type, has an estimated programme cost of $81,283.80 or $ 1.21 per head. Examination alone, without ocular infection swab sampling or testing has an estimated cost of $24,869.64 over the study area, $243.82 per EA or $0.37 per head (data from Table 4).

### Evaluation of test/treat scenarios

**Fig 4:**
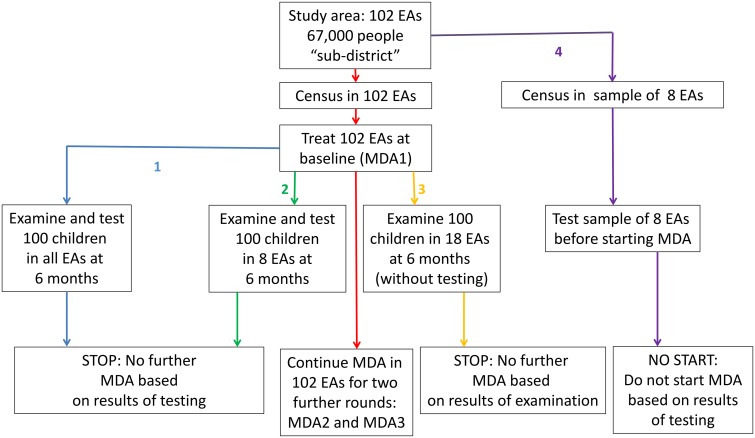
Illustrating alternative strategies involving MDA and testing in the study area (102 EAs 67,156 people) whose costs are compared. In red is the base strategy of three rounds of MDA in the study area. In blue is strategy 1, testing all EAs after one round of treatment. In green, strategy 2, testing 8 EAs after one round of treatment. In yellow, strategy 3, discontinuing treatment based on examination after one round of treatment. In purple, strategy 4 of not starting MDA at all based on prior testing of 8 EAs.

Based on the results of the PRET study, and cost estimates as above we calculated cost estimates for four alternatives to the base strategy of three rounds of MDA throughout the 102 EAs in the study area, which are illustrated in Fig 4. We considered two alternatives directly examined in the PRET study, namely 1) a decision to discontinue MDA in individual EAs based on testing a sample of 100 children within them after the first MDA round, and 2) a decision to discontinue MDA over the whole study area based on testing a subsample of EAs for *Ct* infection. Further, we examined the costs of decisions 3) to discontinue MDA based on demonstrating a reduction in TF below 5% after the first MDA round without laboratory testing and 4) to apply tests for *Ct* infection before embarking on MDA at all. Finally, we considered the effect of the cost of azithromycin in alternatives 1)– 4) if the programme had purchased the drug rather than receiving it from the donation programme.

### Sampling calculations

The PRET baseline EA summarised prevalence of *Ct* infection was 0.8% with a design effect of 4.0, The 6 month post-baseline EA summarised *Ct* prevalence was 0.1% with a design effect of 1.9. For an underlying *Ct* infection prevalence of 0.5–1.0% with these design effects a sample of at most six EAs would provide 95% confidence that the infection prevalence in the study area was less than 5%. Conservatively we assume below that 8 EAs would be an adequate sample. The 6 month post-baseline EA summarised prevalence of TF across all sampled EAs was 2.4% with a design effect of 4.3. For an assumed underlying TF prevalence of 2–3%, approximately 18 EAs would be required to be sampled to demonstrate that TF was less than 5% with 95% confidence.

### Base strategy (census plus 3 MDA rounds-outlined in red in Fig 4)

From the data in Table 2 the estimated cost of census in the study area is $10,639.27 or $0.16 per head. From the data in Table 3 three rounds of standard MDA cost $69,685.38 in the 102 EAs in the study area or $1.05 per head. The total estimated cost of the base strategy applied in the study area is $80,324.75 or $1.20 per head.

### Alternative 1 (discontinue MDA based on testing each EA-outlined in blue in Fig 4)

**Fig 5:**
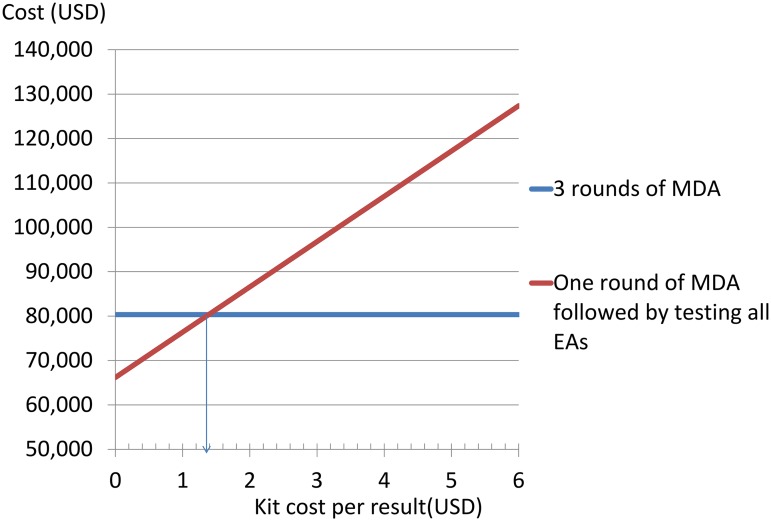
Illustrating how the estimated relative costs of testing 100 children in every EA in the study area (102 EAs, 67,156 people) with an 'Amplicor-like' test after one round of MDA versus continuing MDA in the study area for three rounds depend on the 'kit costs'(cost of obtaining a result).

From the data in Tables 2,3,and 4 the costs of census plus one standard MDA round plus examining and testing 100 children in each EA once is $115,151.63 or $1.71 per head. Thus it costs $34,826.88, $359.50 per EA or $0.51 per head more to test 100 children per EA for *Ct* infection than to treat them annually twice. Fig 5 demonstrates how this depends on the kit cost—in order for testing using an Amplicor-like test to produce savings relative to two unnecessary MDA rounds, a kit cost of $1.38 or less per result would be required

### Alternative 2 (discontinue MDA based on testing a sample of EAs- outlined in green in Fig 4)

In the PRET study, examination and testing was conducted in 48 EAs at an estimated programme cost of $38,163.36 (Table 4). Combining this with census and treatment costs across the study area from Tables 2 and 3 leads to a cost estimate of $72,004.09 or $1.07 per head. Implementation of the district level stopping rules across the study area based on these 48 EAs would have resulted in a saving of $8,320.66, or $0.13 a head. However, following the above sampling calculations, if a sample of 100 children in 8 EAs were examined and tested for *Ct* infection to show that two further MDA rounds were unnecessary in the study area, this would cost $40,242.93 or $0.60 per head and save $40,081.82 or $0.60 a head across the study area.

### Alternative 3 (discontinue MDA based on examining a sample of EAs-outlined in orange in Fig 4)

Following the above sampling calculations, we assume that 100 children in each of 18 EAs could be examined for TF (without testing for Ct infection) to show that two further MDA rounds were unnecessary in the study area. From the data in Tables 2, 3, and 4 this costs $38,256.49 or $0.57 a head, and would save $42,068.26 or $0.63 a head across the study area.

### Alternative 4 (do not start MDA based on tests for infection-outlined in purple in Fig 4)

Based on the above sampling calculations we assume that we could demonstrate that three rounds of MDA were unnecessary through census, sampling and testing in eight of the 102 EAs. From data in Tables 2 and 4 this would cost $7,309.91 or $0.11 per head. Thus, if MDA were not to start at all when baseline infection was less than 5% with 95% confidence, there would be a saving of $73,014.84, or $1.09 per head.

A head to head comparison of alternatives 1 to 4 is given in S1 Table.

### Alternative 5- bought azithromycin

If azithromycin had been bought rather than donated, census followed by three rounds of MDA in the study area would have cost $461,723.05 or $ 6.91 per head (data in Tables 2 and 3). Census followed by one standard round of MDA would cost $161,031.13 or $2.40 per head. This is roughly twice the $1.21 cost per head of testing 100 children for infection in every EA.

## Discussion

We have shown, in the context of the findings of the PRET study, that tests for infection can be applied in trachoma control to prevent further redundant MDA rounds and that in circumstances where an initial MDA round reduces infection below the decision threshold, this will save resources. Using programme costs estimated for The Gambia, discontinuing MDA based on testing 100 children for *Ct* infection in each of a sample of communities, (drawn from a total population of 67,156) after one round of MDA, offers cost savings of $0.57 a head relative to continuing for three MDA rounds, even when the Amplicor test is used. Since PRET found no difference in effectiveness between these study arms, this is also a cost-effective strategy.

The 3^rd^ WHO Global Scientific Meeting on trachoma [17] made recommendations for trachoma surveillance post-MDA based on the sub-district, where a sub-district is a natural or convenient segmentation of a district of 250,000 people. Here we present calculations extrapolated to the whole PRET study area of 102 census EAs and 67,156 people, which despite being made up of four Gambian districts, we believe best corresponds to the ‘sub-district’ envisaged in the recommendations.

We found the average EA treatment cost in The Gambia, based on a single treatment visit to each community not including census was $227.73, which equates to $0.35 per head of population. Enhancing coverage via an extra treatment visit to each community cost a further $0.04 per head, improved coverage in 0–9 year-olds by about 3%, but had no effect on outcome and, in this setting, was not worthwhile. The treatment costs are less than the $ 1.53 per head reported in South Sudan [18], but similar to the $ 0.25 estimate from Mali [19]. A cost of ≤$0.50 per person for trachoma treatment has been quoted in the literature when assessing programme sustainability or cost savings through integrated treatment campaigns targeting several neglected tropical diseases [18,20,21]. Our results are in line with this estimate. The estimated total annual cost of mass treatment with standard coverage and donated azithromycin for the study area (102 EAs and 67.156 people) was $23,224 per round. The main drivers were personnel costs followed by supervision. Others have also found personnel to be the major cost from all cost categories in population-based prevalence surveys [22] and trachoma mass antibiotic treatment distributions [18]. Interestingly, both these studies found transportation to be the next most expensive cost category after personnel, whereas it was not a major cost in our study. This is likely a reflection of the high population density, small distances travelled and relatively good terrain in The Gambia. Costs will likely be higher in countries where the population is sparse and the terrain unforgiving, reflecting increased personnel and transportation costs. This is apparent in the study from the Ayod county of Southern Sudan where a plane had to be chartered to transport personnel [22]. There are different, but not markedly different, costs associated with the variations in EA geography, presumably reflecting different field team organisation, and more hierarchical social structures in smaller settlements, offset by higher transport costs in visiting multiple settlements.

The average cost of testing 100 children per EA for *Ct* infection was $796.90. The major contribution was the kit cost of Amplicor testing at $480.15 per EA. This is less than the cost of 100 Amplicor tests, but more than the cost of 20, because of the strategy of testing in pools of five (requiring 20 tests per 100) and then retesting each individual sample within a positive pool [23], which here reduced costs by over 60% relative to testing each sample individually. We show that, at the EA level, testing using an ‘Amplicor-like’ test would not cost less than two further rounds of MDA unless the kit cost for testing the EA was below $138 per EA or $1.38 per result. However because the number of EAs that would need to be tested to establish district level prevalence with sufficient precision to guide MDA treatment decisions is, in this case, relatively small a district level decision process similar to our ‘stopping rule’ has the potential to save money even with the current costs of testing. The Amplicor test used in this study is no longer commercially available, but alternatives such as the COBAS Amplicor CT/NG Test, Aptima Combo 2 Assay (Gen-Probe Inc. CA, USA), and the Real-time CT/NG Assay (Abbott Laboratories, Illinois, USA) platform are no cheaper and some [24] but not all have had pooling strategies validated. Nevertheless, this study suggests that even the application of relatively costly Nucleic Acid Amplification Tests (NAATs) may save resources if a sample of representative communities was tested for infection before MDA was implemented

The ‘stopping rule’ used in PRET was an arbitrary one based on the observation that if no infections were found in a sample of 100 children then there was 95% confidence that the true infection prevalence was less than 5%. Whether an infection prevalence of 5% in 0–5 year-olds is the best cut-off for discontinuing MDA is unknown. It has been pointed out by others that studies to ascertain a threshold or thresholds below which trachoma infections do not persist and will then disappear on their own (i.e. the existence of an ‘Allee effect’) will be very difficult to conduct against the background of worldwide downward secular trends in trachoma prevalence [25]. However, one interpretation of the PRET data would be that The Gambia reached such a point before the PRET study started, when the application of tests for infection would have shown a baseline infection prevalence of 0.8%, The potential we outline for a testing strategy to save money is likely to vary with both TF prevalence and Ct infection rates and with the number of children tested in a population unit. Here TF prevalences in the 5–10% range were compatible with extremely low rates of *Ct* infection, but the extent to which this is generalisable needs to be established in other studies in which district or sub-district level TF and *Ct* infection rates are both estimated. In PRET, 100 children per EA were tested as if there were zero cases of infection there was 95% confidence that the true rate in the EA was less than 5%. For a district rather than EA based MDA decision fewer children per EA could be tested and our data could be extrapolated to estimate costs in those circumstances. We suggest that tests for *Ct* infection will be needed to confirm whether, or, more likely, when a country, region or district is ready to discontinue MDA. We show that the application of such tests with appropriate sampling schemes and decision rules will save money relative to initiating or continuing MDA, even with the current costs of NAATs.

Our study had a number of limitations which may affect the results. We did not include the opportunity cost of people coming to be treated and/or examined in relation to what they would otherwise have been doing, what this represented in monetary terms, and how long each adult spent with the team. The NEHP workers administering treatment were taken away from their usual tasks, also incurring societal costs. Our total cost estimates are therefore likely to under-estimate the total societal cost. By not including the opportunity cost, we may have masked cost differences between the strategies, depending on the amount of time an adult spent when attending and accompanying children for treatment distribution, compared with accompanying children for examination. Studies comparing different trachoma treatment strategies have noted that patient (opportunity) cost is a major cost in mass azithromycin treatment with donated drug [19,26]. We probably under-estimated the cost of the extra treatment day in the enhanced treatment strategy as, per person, the teams would have spent longer per person treated trying to find the few remaining individuals needing treatment than treating people when arriving in an EA for the first day. As the extra day had no impact on outcome, this concern is minor. Finally, however, our data are taken from a research project. Although we have made substantial efforts to separate out and remove research costs, the census, treatment and examination activities may have been organised and conducted differently if entirely planned and run by the programme. Programmes may elect for example to combine census and treatment activities. Thus, absolute costs would probably vary with a different project organisation (and be different in another low prevalence country), but we suggest that the relative cost differences we highlight between three annual MDAs and a stopping rule based on testing for infection would be less affected.

If azithromycin were purchased rather than donated, the cost of treatment was 6.7 times greater with azithromycin constituting over 80% of the total cost. This is much more than the costs of testing and, without the donation programme it would be cheaper to test first if there were even a minority of communities with low levels of infection as purchasing azithromycin is prohibitively expensive [19,26–28] This underlines the importance of the donation to national eye health programmes in their quest to eliminate trachoma as a public health problem.

## Conclusion

We have shown that the strategy of three annual rounds of mass azithromycin treatment of a sub-district are more expensive than examining and testing ocular swabs from a sample of EAs, if, as in the PRET study treatment can then be discontinued based on the results after one round. Therefore, in low prevalence settings, it could be both cost-saving and cost-effective to implement a stopping rule strategy.

## Supporting Information

S1 Table Spreadsheet with calculations of costs of test/treat scenarios (XLS)

S1 Checklist STROBE checklist. (DOC)
